# Inference of partial colexifications from multilingual wordlists

**DOI:** 10.3389/fpsyg.2023.1156540

**Published:** 2023-06-16

**Authors:** Johann-Mattis List

**Affiliations:** ^1^Department of Linguistic and Cultural Evolution, Max Planck Institute for Evolutionary Anthropology, Leipzig, Germany; ^2^Chair of Multilingual Computational Linguistics, University of Passau, Passau, Germany

**Keywords:** partial colexification, loose colexification, colexification networks, computational comparative linguistics, computer-assisted language comparison

## Abstract

The past years have seen a drastic rise in studies devoted to the investigation of colexification patterns in individual languages families in particular and the languages of the world in specific. Specifically computational studies have profited from the fact that colexification as a scientific construct is easy to operationalize, enabling scholars to infer colexification patterns for large collections of cross-linguistic data. Studies devoted to partial colexifications—colexification patterns that do not involve entire words, but rather various parts of words—, however, have been rarely conducted so far. This is not surprising, since partial colexifications are less easy to deal with in computational approaches and may easily suffer from all kinds of noise resulting from false positive matches. In order to address this problem, this study proposes new approaches to the handling of partial colexifications by (1) proposing new models with which partial colexification patterns can be represented, (2) developing new efficient methods and workflows which help to infer various types of partial colexification patterns from multilingual wordlists, and (3) illustrating how inferred patterns of partial colexifications can be computationally analyzed and interactively visualized.

## 1. Introduction

The past years have seen a drastic rise in studies devoted to the investigation of colexification patterns in individual languages families and the languages of the world. The concept of *colexification* has proven specifically useful for computational and quantitative approaches in lexical typology. The term *colexification* was originally proposed by François (2008) as a cover term for all cases where multiple senses are expressed by one word form, no matter whether the multitude of senses results from polysemy or homophony. Colexifications can be easily computed from large collections of lexical data, specifically from multilingual wordlists, in which a certain number of concepts is translated into several target languages (see List, 2014, p. 22–24). Through the aggregation of several multilingual wordlists, it is straightforward to assemble large amounts of cross-linguistic colexification data, as witnessed by the growth in recent versions of the Database of Cross-Linguistic Colexifications (CLICS; List et al., 2018; Rzymski et al., 2020; https://clics.clld.org), as well as by the increase in studies which exploit colexification data assembled from different sources (Bao et al., 2021; Di Natale et al., 2021). Quantitative studies on colexification patterns have also shown that it is straightforward to extract those colexifications that are most likely to result from polysemy by searching for colexifications recurring across several language families— as opposed to frequent colexifications inside one and the same language family, wich might reflect wide-spread cases of homophony (List et al., 2013). This means in turn that large colexification networks can be treated as polysemy networks that give us direct insights into certain aspects of lexical semantics (Youn et al., 2016; Jackson et al., 2019; Harvill et al., 2022).

Up to today, however, most studies dealing with colexifications focus on colexifications of *entire words*. Colexifications involving only certain parts (i.e., *morphemes*) of the words in a given language — *partial colexifications*, also called *loose colexifications* (François, 2008)—have rarely been investigated (see Urban, 2011 for an exception) and rarely been computed automatically from larger collections of cross-linguistic data (see List et al., 2022 for initial attempts). As an example for *loose* or *partial colexification* in the sense of François (2008, p. 171), consider the words English *straight* and *straightforward*, which share the morpheme *straight* and thus *loosely colexify* the two senses “rectilinear” and “simple.” Two major factors seem to account for the problems involving studies with partial or loose colexifications. On the one hand, it is less straightforward to model partial colexifications in networks, since the relations between words that share common parts may at times be asymmetric, with one word being entirely repeated in the other word. Not only are different network types needed to model partial colexification networks, it is also much less straightforward to interpret them. On the other hand, it is difficult to *infer* partial colexifications networks from large collections of cross-linguistic data, since partial commonalities between words easily arise by chance or reflect grammatical distinctions (noun classes, gender marking, and part of speech). As a result, a method that naively searches for similarities between words in the same language variety in a large corpus typically provides very densely connected noisy networks in which one barely finds any signal that would be interesting from a semantic or cognitive perspective. Thus, while it is easy to handle noise due to homophony in the case of full colexification networks by using strict thresholds for the occurrence of particular colexifications in combination with normalized weights, it is difficult to use the same criteria when creating partial colexification networks.

This study attempts to address at least some of these problems by proposing new models with which certain kinds of partial colexification patterns can be represented in networks, and by developing new efficient methods and workflows that help to infer different types of partial colexification patterns from multilingual wordlists. Having inferred these patterns, the study further shows how they can be visualized and analyzed.

## 2. Materials and equipment

### 2.1. Multilingual wordlists

The starting point of our new workflow for the inference of partial colexifications are multilingual wordlists. A wordlists is hereby understood as a collection of word forms which are arranged by their meaning. Unlike a dictionary, in which the word form (the *headword*) constitutes the primary linguistic unit by which data are ordered, a wordlist orders words by their meaning. While a dictionary starts from the form, following a semasiological or form-based perspective, a wordlist starts from the meaning, following an onomasiological, or concept-based perspective. As a result, a multilingual wordlist allows us to compare how certain *concepts* (which are thought to be generally comparable across languages, even if this may be problematic in practice) are translated into certain languages.

The compilation and aggregation of multilingual wordlists has seen remarkable progress during the last decade and the number of digitally available wordlist collections is constantly increasing. On the one hand, large unified multilingual wordlist collections have been published in the past years (Haspelmath and Tadmor, 2009; Key and Comrie, 2016; Dellert et al., 2020), on the other hand, standards for cross-linguistic data formats have been constantly improved (Forkel et al., 2018) and applied to many smaller or growing data collections (Ferraz Gerardi et al., 2021) and for the purpose of *retro-standardization* (Geisler et al., 2021).

### 2.2. Cross-Linguistic Data Formats

For the exploration of partial colexification patterns across multiple languages, a modified version of the well-known *Intercontinental Dictionary Series* (IDS) was prepared (Key and Comrie, 2016). While the original version mixes phonetic transcriptions with language-specific phonological transcriptions and orthographic entries, the entries in the modified version were semi-automatically converted to the International Phonetic Alphabet in the variant proposed by the Cross-Linguistic Transcription Systems (CLTS) reference catalog (https://clts.clld.org; List et al., 2023; see Anderson et al., 2018). The conversion was done by applying the Lexibank workflow of creating standardized wordlists in Cross-Linguistic Data Formats (List et al., 2022). In this workflow, originally non-standardized datasets are semi-automatically standardized by applying a mix of software tools (based on CLDFBench; Forkel and List, 2020) and manual annotation in order to convert the data into the formats recommended by the Cross-Linguistic Data Formats initiative (Forkel et al., 2018).

The updated version of the IDS provides wordlists for 329 language varieties for up to 1,310 concepts. The standardized phonetic transcriptions consist of a total of 558 distinct sounds (types) which occur 2,902,306 times in the data (tokens), with an average phoneme inventory size of 50.76 sounds per variety. Although—strictly speaking—partial colexifications could in theory also be identified from orthographic data, being able to work with a larger multilingual wordlist available in phonetic transcriptions has two major advantages, even if the transcriptions may contain certain errors. First, it is easier to evaluate the findings if transcriptions are harmonized; secondly, knowing that sounds are represented in segmented form makes it easier to select the thresholds by which partial colexifications are preliminarily accepted or discarded. The revised version of the Intercontinental Dictionary Series is currently curated on GitHub, where it can be accessed at: https://github.com/intercontinental-dictionary-series/ids-segmented. The version used in this study is v0.2 (https://github.com/intercontinental-dictionary-series/idssegmented/tree/0.2).

For developmental purposes and in order to test certain technical aspects of the new methods proposed here, a smaller wordlist by Allen (2007) was used. This list—also converted to CLDF (see: https://github.com/lexibank/allenbai)—offers data for nine Bai dialect varieties in standardized phonetic transcriptions.

## 3. Methods

Full colexifications across languages can be handled in an efficient way that has shown to provide very interesting insights into semantic relations. Partial (or loose) colexifications, however, suffer from noise, resulting from the fact that partial similarities between words in the same language may result from a large number of factors (coincidence and grammatical markers) that do not reflect specific semantic relations between the words in question. As a result, the well-established workflows for the inference of full colexification networks cannot be used to infer partical colexification networks. In order to handle this problem, I propose a three-stage approach that starts from the *modeling* of partial colexifications—which helps to reduce the search space and provides a consistent representation of distinct types of partial colexifications in networks—, offers efficient methods for the *inference* of specific partial colexification types, and finally allows us to *analyze* different kinds of partial colexification networks in various ways. In this context, modeling, inference, and analysis reflect a general approach to scientific problem solving in the historical sciences that was inspired by its application in evolutionary biology (Dehmer et al., 2011).

### 3.1. Modeling partial colexifications across languages

#### 3.1.1. Major types of partial colexification

When modeling words as sequences of sounds, we can define major sequence relations in a formal way. Since sequences play a crucial role in many scientific fields—ranging from computer science (Gusfield, 1997) via bioinformatics (Durbin et al., 2002[1998]) to physics (Kruskal and Liberman, 1999[1983])—basic relations between sequences have been independently identified and discussed long ago. In the following, we will distinguish the term *partial colexification* from the term *loose colexification* (the latter originally termed by François, 2008). According to this distinction, partial colexifications are restricted to those cases where two word forms, modeled as sound sequences, share at least one non-grammatical morpheme that has the same surface form. Following the examples provided by François (2008), loose colexifications would then refer to all cases where two word forms that express different senses share at least one non-grammatical *cognate* morpheme. According to this distinction, English *old* and its comparative form *older* can be said to colexify partially (and loosely), while German *alt* “old” and its comparative ā*lter* “older” would only colexify loosely. The narrower notion of partial colexifications has the advantage that we can use existing models and insights from earlier studies on sequence and string relations and adopt them to the notion of partial colexifications.

When comparing three fictitious sequences ABC, XYABCD, and ZABCEF, it is easy to see that the first sequence ABC recurs in both the second and the third one. In computer science and bioinformatics, ABC is called a *common substring* of XYABCD and ZABCEF. Since there is no longer common substring than ABC, it is furthermore the *longest common substring* between both sequences. Regarding the specific relation between XYABCD and ZABCEF, we can say that they share a common substring of length 3. The sequence ABC also shares substrings of length 3 with the two other sequences ZABCEF and XYABCD. In addition, however, we can see that the sequence ABC is *identical* with the common substring, and we can say that ABC is a part of XYABCD and ZABCEF. While the common substring relation between sequences is commutable, the part-of relation isn't: saying that one sequence A is part of another sequence B is not the same as saying that sequence B is part of sequence A.

Given their importance for a wide range of scientific and industrial applications, many efficient algorithms for the inference of common substrings and the identification of part-of relations (or parthood relations; see Hoehndorf et al., 2009) in sequences have been proposed (see the overview in Gusfield, 1997, p. 89–121). Common substring relations and part-of relations are two fundamental relations between sequences. Partial colexifications, in the sense define above, reflect a specific subtype of these relations, in so far as words sharing at least one non-grammatical morpheme with a common surface form also share a common substring and could also reflect a part-of relation.

#### 3.1.2. Affix and overlap colexifications

When trying to develop methods that search for partial colexifications from multilingual wordlists, we have the problem that the segmentation of words into morphemes is usually not given to us. While it would sure be possible to annotate morphemes manually, this would require a detailed expert knowledge of the languages in question that we often do not have, not to speak of the amount of time and labor it would cost to provide these annotations for hundreds of languages. Instead of carrying out the segmentation of words into morphemes directly, we can alternatively search for common substring and part-of relations across individual languages. This will result in many matches that do not reflect true non-grammatical morphemes, but we may hope to handle the noise when considering a large enough amount of languages from different families and areas. In order to further facilitate the search for partial colexifications, it is additionally useful to restrict the search space by working with a narrower notion of shared similarity than the one reflected in the common substring and the part-of relation between sequences. Thus, instead of searching whether a word form A expressing a concept X is part of a word form B expressing a concept Y, we can ask if A is identical with the beginning or the end of B, or—to put it in other terms—, if A is a *prefix* or a *suffix* of B (in the sense in which *prefix* and *suffix* are used in computer science). Similarly, we can ask if A and B *overlap*, that is, if they share a common substring which is either a prefix or a suffix of both sequences.

The search for these potential affix and overlap colexifications can be further restricted by setting thresholds for the length of the substring which the sequences should share, and by setting thresholds for the length of the remaining parts of the sequences which they do *not* share. Here, the fact that our multilingual wordlist is now available in the form of fully segmented, standardized phonetic transcriptions, comes in handy, since it allows us to set up thresholds for a certain number of *sounds* rather than a certain number of *symbols* which often reflect individual sounds only in combination.

#### 3.1.3. Representing partial colexifications in networks

Affix and overlap colexifications, as they were introduced before, are special cases of the part-of and the common substring relation between sequences. Like part-of relations, affix colexifications entail a *directional relation* (one sequence is a part of the other sequence, in this specific case, appearing in the beginning or the end). Like common substring relations, overlap colexifications do not entail a directional relation.

In contrast to the well-known undirected weighted network models used for the representation of full colexification networks (List et al., 2013) which can be easily visualized, both interactively (Mayer et al., 2014) and statically (using edge thickness to account for differences in the weights for the links connecting individual concepts, see List et al., 2018), weighted *directed* networks draw a link from one concept A to another concept B only in those cases where an affix colexification from A to B can be attested. As an example, consider the words German *Finger* “finger” and *Fingernagel* “fingernail.” Here, the word *Finger* is an affix (in the sense in which affix is used in computer science) of the word *Fingernagel*, and we can therefore draw a link from the concept “FINGER” to the concept "FINGERNAIL” in an directed network. When visualizing these networks, we may have links pointing in both directions. While we would not necessarily expect links for “finger” and “fingernail” to go in both directions, this may happen with other concept pairs, depending on the underlying lexical motivation.

**Figure 1:**
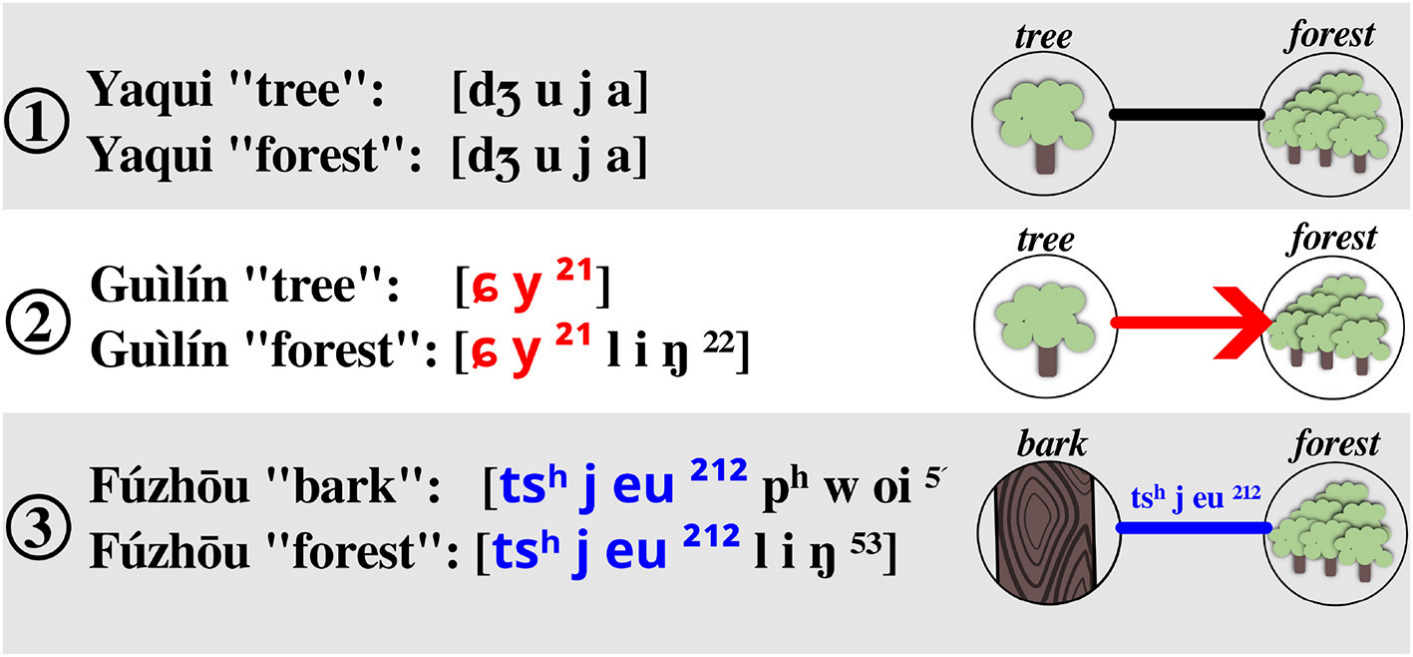
Overview of three major colexification types discussed in this study. (1) Provides an example for a full colexification in Yaqui (data from CLICS^3^ Rzymski et al., 2020), (2) shows an example for the directional representation of affix colexifications with an example from Guìlín Chinese (data from Liú et al., 2007), and (3) shows an example for overlap colexification in Fúzhōu Chinese (data from Liú et al., 2007).

Overlap colexifications can be handled in the same way in which one would handle full colexifications. The difference is that one should internally store the individual suffixes that make up for the overlap connection. Thus, while it is enough to store one word form for a colexification in a full colexification network (since words colexifying two or more concepts are by definition identical), it is important—for the sake of transparency—to indicate the actual suffix that recurs across two words in an overlap colexification network. Figure 1 contrasts the three types of colexification networks along with some simplified examples.

### 3.2. Efficient inference of full and partial colexification networks

A naive implementation of a simple search for partial colexifications (be they affix or overlap colexifications) would take all word forms from one language and then compare each word against each other word in the sample, storing observed commonalities. While this procedure is easy to understand and certainly yields the desired results, it is far away from being efficient. As a result, specifically when dealing with large cross-linguistic data collections, it is advisable to use efficient search strategies.

**Figure 2:**
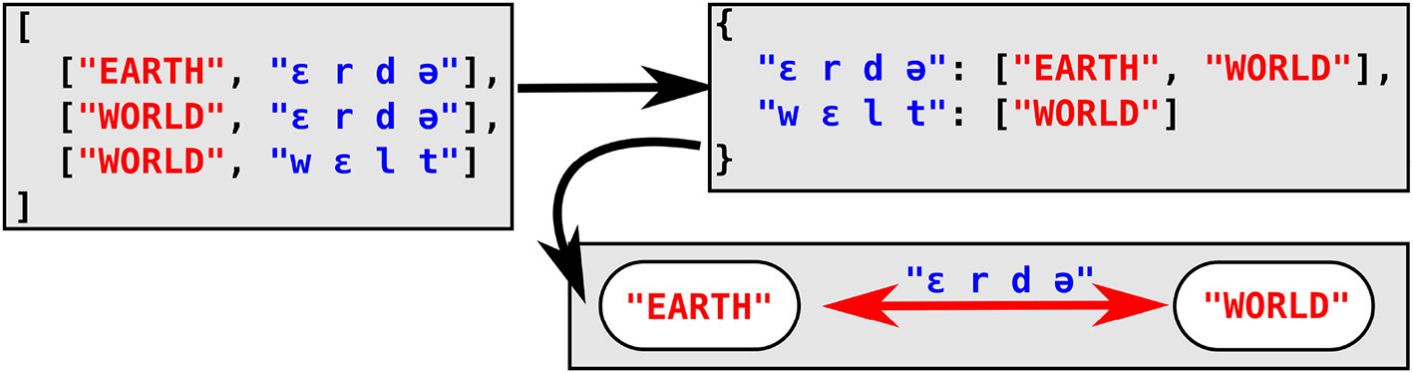
Efficient search for full colexifications using associative arrays. Data are represented in JSON format for a wordlist consisting of three German word forms *Erde* “EARTH,” *Erde* “WORLD,” and *Welt* “WORLD.” The top-left box shows the initial format of the data (a wordlist consisting of two columns, one storing the concept and one storing the word form in IPA. The top-right box shows the resulting associative array, in which the forms serve as a key and concepts expressing this form are added to the same array as a value. The bottom-right box shows the resulting colexification inferred from this example.

For the computational of full colexifications, an efficient search strategy consists in the use of *associative arrays* as a major data structure, which consists of a key that allows to access a given value. In the Python implementation used by the CLICS database (List et al., 2018; Rzymski et al., 2020), the keys consist of the individual word forms for a given language, while the values are a list of the concepts that the form links to. In order to infer colexifications for a given language, the method iterates over all words for a given language in a wordlist and subsequently adds them to the associative array, storing the concept that the word form expresses in the list that constitutes the value of the associative array. If a given word form has already been added to the array, the associated list is expanded by adding the new concept in question. In a second stage, the method iterates over all keys in the associative array and adds all pairs of diverging concepts in the list to the growing network of colexifications across several languages. Detailed descriptions of this procedure can be found in a tutorial accompanying Jackson et al. (2022) and in List (2022). Figure 2 shows the structure resulting from applying this method to a small wordlist of three German words.

**Figure 3:**
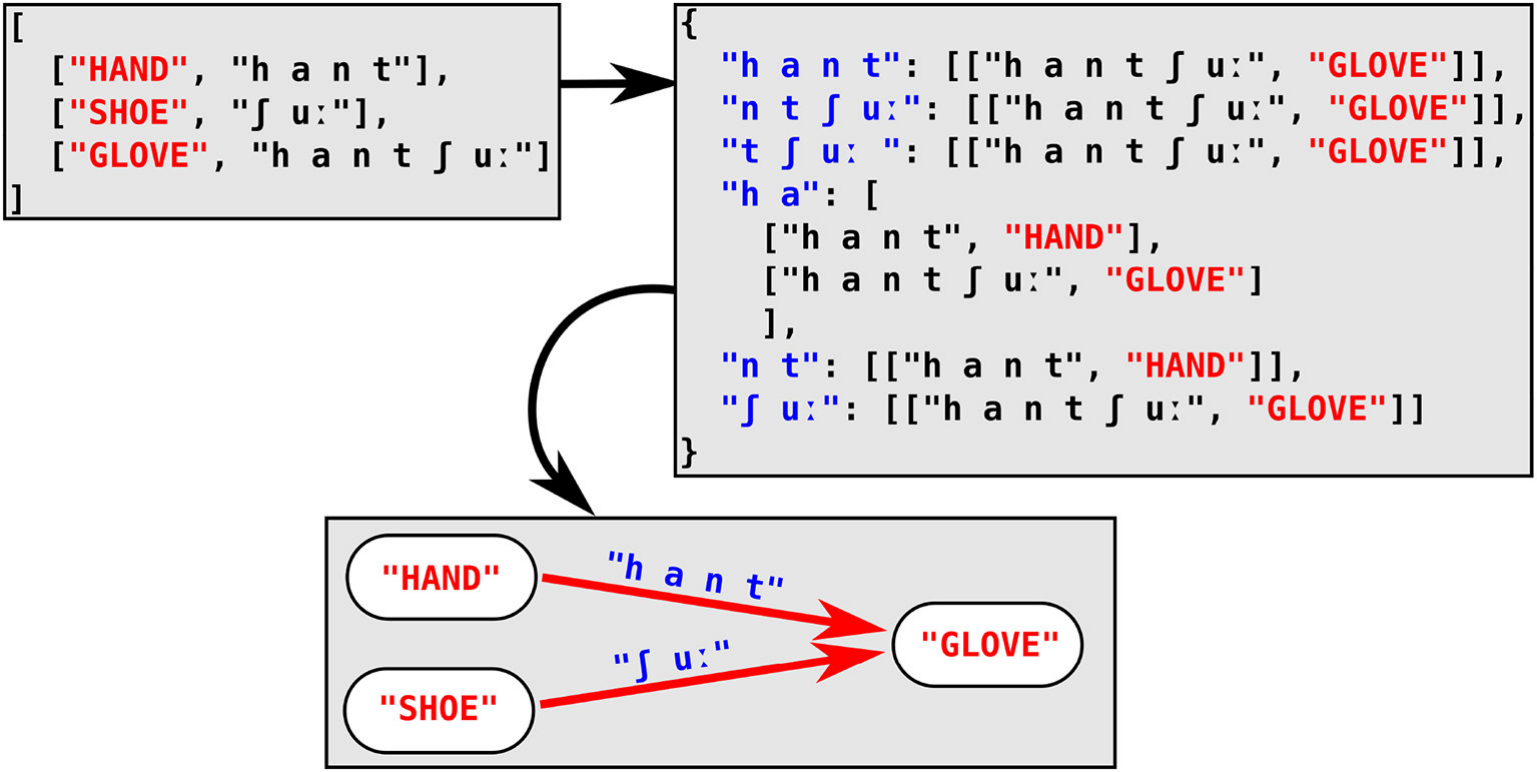
Efficient search for affix colexifications, illustrated for a wordlist of three German words *Hand* “HAND,” *Schuh* “SHOE,” and *Handschuh* “GLOVE” (lit. “hand-shoe"). Starting from the original wordlist in the top-left box, each word form is represented by all possible prefixes and suffixes that match the two-threshold criterion (see text) in the associative array in the top-right box. When iterating over the word forms in the original concept list, we find that two words, *Hand* and *Schuh* are stored in the array and we can therefore infer an affix relation between the two words and the word *Handschuh*, represented in the form of a directed graph in the box at the bottom of the figure.

In our approach to partial colexifications, we proceed in a similar fashion, by iterating over the wordlist of each individual language twice. In order to find potential affix colexifications, however, the associative arrays are filled with affixes of varying size, and the list serving as the value is then filled with tuples of the corresponding full word form and its concept. The affixes are computed by iterating from the left and the right of the sound sequence representing the word form. Affix sizes are limited by two thresholds. The first threshold (default set to 2) limits the minimum size of the affix to 3 sounds. The second threshold makes sure that the size of the remaining word part is larger than a certain minimum (default set to 2). In combination, both thresholds guarantee that the affix we infer has a reasonably large size, and that the full word form to which we link it is also large enough to increase the chances that we detect compounding structures rather than cases of inflection. With these thresholds, we can detect all potential *affix candidates* for a given word in a first run and store them in our associative array. In a second step, we then iterate over all original words in the data, sorted by length, starting with the longest word. For each of the word forms, we then check if it occurs in the array of affix candidates. If this is the case, this means that the word appears as the affix of one of word forms linked as a value to this array, and we can add them to our network, by adding a link from the word recurring as affix to the word containing the affix (see Figure 3).

For the identification of overlap colexifications, we pursue the same strategy as for affix colexifications in the first stage, by populating an associative array with affix candidates for the word forms in our wordlist. Due to the increase in noise when searching for overlap colexifications the default thresholds for the length of the affix are set to 4 and the threshold for the length of the remaining part are set to 3. In the second stage, we iterate over the array with affix candidates itself, which has been sorted by the length of the affixes serving as keys in reverse order (starting from the longest affix found in the data for a given language). For each affix, we then compare all word pairs in which this affix recurs and check that neither of the two forms appears as a suffix or a prefix of the other form. If these conditions are met and the forms are also not identical (which would correspond to a full colexification), we store the forms as overlap colexifications along with the affix by which the forms overlap.

As can be easily seen from the descriptions, the complexity of the three methods for the inference of full colexifications, affix colexifications, and overlap colexifications differs. The search for full colexifications requires the least amount of computation time, followed by the search for affix colexifications, and by the search for overlap colexifications.

With the methods for the inference of full and partial colexifications in individual languages above, we can construct full and partial colexifications networks by applying the search strategies to multiple languages and iteratively growing a colexification network, in which we add edges when new edges are inferred for a particular language, or increase edge weights when edges have been already attested during the iteration. The networks computed in this form are all annotated in various ways. For the nodes, we store the number of word forms that can be found in the data, the number of language families in which these words are attested, and the actual word forms in each language. For the links between the nodes, we store the number of concrete word forms which exhibit the colexification relation, the number of language families, in which these colexifications can be found, and the actual word forms (including the colexifying parts for partial colexifications) in which the colexifications occur. For affix colexifications, we infer a directed network, while the network for full and overlap colexifications is undirected.

### 3.3. Analyzing partial colexification networks

In order to understand major differences between full colexification networks and the two new types networks introduced here, one can compare their *degree distributions*. The degree of a node in a network is the number of its edges (Newman, 2010, p. 133–135). The weighted degree of a node in a network is the sum of the edge weights of its edges. While we have only one type of degree for undirected networks, we have two possible degrees for network with directed edges, the *in-degree* and the *out-degree*, with the former representing the number (or the sum of the edge weights) of incoming edges of a given node, and the latter representing the number (or the sum of the edge weights) of outgoing edges of a given node in the network. In order to compare the degree distributions of two networks constructed from the same set of nodes, we can compute the Spearman rank correlation (Spearman, 1904), which tell us to which degree those nodes that show a very high degree in one network also show a high degree in the other network, and to which degree nodes with low degrees in one network also tend to show low degrees in the other one.

In addition to the comparison of degree distributions, it is also useful to visualize the networks and to zoom in to interesting parts that illustrate where major differences can be found. This can be done quite conveniently now with the help of software packages for network visualization, such as Gephi (Bastian et al., 2009) or Cytoscape (Shannon et al., 2003; Smoot et al., 2011). For the visualizations reported here, Cytoscape is used.

### 3.4. Implementation

The methods reported here are implemented in Python and shared in the form of Python library that can be used as a plugin to the CL Toolkit package (https://pypi.org/project/cltoolkit; List and Forkel, 2021). CL Toolkit was designed to allow to access CLDF Wordlists that conform to the standards proposed by the Lexibank repository (List et al., 2022) conveniently from Python scripts or from the Python interactive console. For the handling of graphs, the NetworkX package was used (Hagberg et al., 2008), and for the inference of communities, the Igraph package was used (Csárdi and Nepusz, 2006). The computation of rank correlations was done with SciPy (Virtanen et al., 2020). The Supplementary material offers access to all data and code necessary to replicate the results reported here.

## 4. Results

### 4.1. Computation time of efficient colexification inference

In order to test whether the newly proposed method for the inference of affix colexifications is indeed more efficient than a conceptually much simpler comparison of all word against all words in a word list, a small experiment was designed in which the CLDF dataset of Bai dialects derived from Allen (2007) was analyzed several times and computation times were calculated. The results of this test indicate that the new method is indeed much more efficient in terms of computation time than the naive iteration. In various experiments on different Linux machines, computation time differences show that the naive all-to-all word comparison needs more than five times as much time than the new efficient approach, while both produce exactly identical results (detailed examples on this test are provided in the Supplementary material accompanying this study). While computation time may be less important when working with small datasets of only about a dozen of languages, it can become a bottleneck when working with large datasets such as the Intercontinental Dictionary Series. For this reason, the efficient solution proposed here, is proving very useful. This does not mean, however, that the solution is perfect, and it may well be the case that there are more efficient solutions available (e.g., using *suffix trees*, see Gusfield, 1997, p. 122–180) that could be implemented in the future.

### 4.2. Comparing degree distributions

Having computed colexification networks for full colexifications, affix colexifications, and overlap colexifications, the Spearman rank correlation was computed for the weighted degree distributions of all three colexification types, splitting affix colexifications into two types of degree distributions, the in-degree and the out-degree. The results of this comparison are given in Table 1. As can be seen from this table, two moderate correlations can be observed for the total of six pairings. The degree distribution of the full colexification network correlates moderately with the out-degree distribution of the affix colexification network (ρ = 0.50, *p* < 0.0001), and the degree distribution of the overlap colexification network correlates moderately with the in-degree distribution of the affix colexification network (ρ = 0.42, *p* < 0.0001).

**Table 1:** Comparing the Spearman rank correlations for the four different kinds of degree distributions.

**Colexification type A**	**Colexification type B**	**Nodes**	**ρ**	***P*-value**
Full colexification	Affix colexification (in-degree)	1,308	0.0960	<0.0001
Full colexification	Affix colexification (out-degree)	1,308	0.5034	<0.0001
Full colexification	Overlap colexification	1,307	0.1179	<0.0001
Affix colexification (in-degree)	Affix colexification (out-degree)	1,308	–0.0830	<0.0001
Affix colexification (in-degree)	Overlap colexification	1,307	0.4212	<0.0001
Affix colexification (out-degree)	Overlap colexification	1,307	–0.0488	0.0104

As can be seen, we can observe significant moderate correlations for Full Colexifications as compared to the out-degree of affix colexifications (0.5) and for the in-degree of affix colexifications compared to overlap colexifications (0.42). For the other pairings, no significant correlations can be observed.

Interpreting these results may not seem straightforward at the first sight, and additional research will be needed to confirm the explanations given in the following, but it seems to me that both correlations reflect two properties of concepts: *lexical root productivity* and *compoundhood*. The correlation between the weighted degree of concepts in full colexification networks and the out-degree of concepts in affix colexification networks points to a tendency according to which concepts that are often fully colexified with other concepts *also* seem to be frequently *reused* as compounds or affixes in complex words. While this finding may seem to be quite reasonable or even obvious, it was so far not possible to confirm it in cross-linguistic studies. Partial colexification networks thus point us to an important property of concepts that tend to colexify frequently across the languages in the world: their propensity to be reused in word formation processes to form new words. This property, which I propose to call *lexical root productivity* (the term is inspired by a discussion with Alexandre François; see List, 2019a,b), plays a key role in lexical motivation, the process underlying the formation of new word forms in the languages of the world (Koch, 2001).

The correlation between the weighted degree distribution of overlap colexifications with the out-degree distribution of affix colexifications has an even more straighforward explanation. Concepts that exhibit many overlap colexifications across a larger sample of languages are concepts that are often expressed with the help of compounds or morphologically complex words. The same holds for those concepts that have many incoming edges in an affix colexification network. As a result, the correlation between the two degree distributions is not very surprising. It shows, however, that both the weighted in-degree of affix colexification networks and the weighted degree of overlap colexification networks can be used as a proxy to measure the *compoundhood of concepts* (a term inspired by Martin Haspelmath, p. c.), that is, the tendency of concepts to be expressed by compound words or morphologically complex words.

### 4.3. Inspecting colexifications through subgraphs

While the investigation of the degree distributions already gives us a nice impression about the commonalities and differences between different kinds of colexification networks, a closer investigation of smaller parts of the graphs can help us to see these differences much more clearly. In order to provide a fruitful sample, the Infomap algorithm (Rosvall and Bergstrom, 2008) was used to compute communities from the full colexification network. In a second step, 23 concepts which show different properties with respect to their full and partial colexifications, were selected and the corresponding subgraphs for full, affix, and overlap colexification networks were extracted and visualized with the help of Cytoscape (Shannon et al., 2003; Smoot et al., 2011).

**Figure 4:**
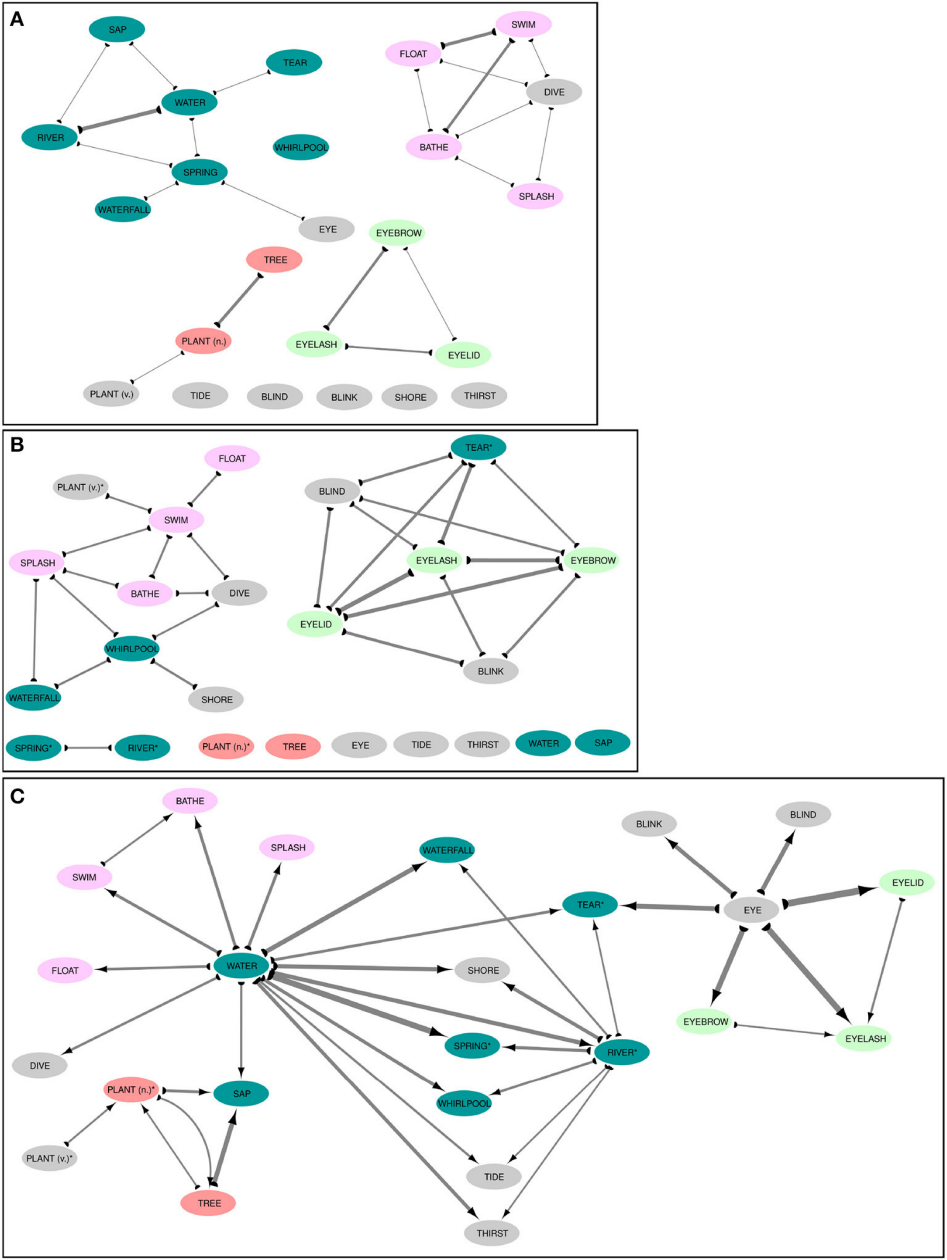
Comparing full **(A)**, overlap **(B)**, and affix **(C)** colexifications for subgraphs of the IDS dataset. Line width indicates the weight of the colexifications, colors other than light gray indicate communities inferred for the full colexification network, and link directions in the affix colexification network **(B)** are displayed with the help of arrows. Concept labels are taken from the Concepticon project. Concept labels with an asterisk were modified to to enhance the visualization.

As can be seen from the visualizations shown in Figure 4, the three networks show a remarkable difference in their individual structures, although they all involve the same concepts. Thus, while the concept EYE has only one spurious link in the full colexification network to SPRING (A), it is completely isolated in the overlap colexification network (B), while appearing as a rather central concept with a high out-degree in the affix colexification network (C). When inspecting connected components in all three networks, we find huge differences between the concepts that are fully connected with each other, while it is easy to spot semantic or morphological connections that give rise to these patterns. Thus, we find a cluster of BLIND, TEAR, EYELASH, EYEBROW, EYELID, and BLINK in the overlap colexification network that clearly seems to result from the fact that the words expressing these concepts all contain a morpheme for EYE. The central position of EYE in the affix colexification network confirms this role, and we find similar structures for WATER as another central concept in the affix colexification network. A systematic comparison of these different kinds of colexification networks allows us to identify semantic *key players* (Lee et al., 2020) that play an important role in contributing morphological material to the construction of the lexicon of many of the world's languages.

## 5. Discussion

This study has presented new ideas regarding the inference of partial colexification networks from multilingual wordlists. It has introduced new models that can be used to handle partial colexification patterns and proposed new efficient methods and workflows for the inference of partial colexification networks. Two new models for colexification networks were introduced, namely *affix colexification networks* and *overlap colexification networks*. Using these new types of colexification patterns to infer affix and overlap colexification networks from large multilingual wordlist revealed some interesting properties of both network types. While overlap colexification networks allow us to measure the *compoundhood* of individual concepts across the world's languages, affix colexification networks could be used as an initial proxy to measure *lexical root productivity* across languages. Apart from being interesting for people working in the field of lexical typology, there is hope that these new types of colexification networks can be very useful for many additional scientific fields in the future, including most notably computer science (and approaches to computational semantics) and psychology.

## Data availability statement

Data and code needed to replicate the experiments presented in this study are curated on GitHub (https://github.com/lingpy/pacs/releases/tag/v1.0) and archived with Zenodo (https://doi.org/10.5281/zenodo.7980852).

## Author contributions

J-ML designed the study, wrote the code, prepared the data, and wrote the text.
